# Cardiac Troponins are Among Targets of Doxorubicin-Induced Cardiotoxicity in hiPCS-CMs

**DOI:** 10.3390/ijms20112638

**Published:** 2019-05-29

**Authors:** Michaela Adamcova, Veronika Skarkova, Jitka Seifertova, Emil Rudolf

**Affiliations:** 1 Department of Physiology, Faculty of Medicine in Hradec Kralove, Charles University in Prague, Simkova 870, 500 03 Hradec Kralove, Czech Republic; 2 Department of Biology, Faculty of Medicine in Hradec Kralove, Charles University in Prague, Zborovská 2089, 500 03 Hradec Kralove, Czech Republic; HanusovaV@lfhk.cuni.cz (V.S.); seifertovaj@lfhk.cuni.cz (J.S.); rudolf@lfhk.cuni.cz (E.R.)

**Keywords:** hiPCS-CMs, doxorubicin, cardiotoxicity, morphology, troponins, mitochondria

## Abstract

Modern diagnostic strategies for early recognition of cancer therapeutics-related cardiac dysfunction involve cardiac troponins measurement. Still, the role of other markers of cardiotoxicity is still unclear. The present study was designed to investigate dynamics of response of human cardiomyocytes derived from induced pluripotent stem cells (hiPCS-CMs) to doxorubicin with the special emphasis on their morphological changes in relation to expression and organization of troponins. The hiPCS-CMs were treated with doxorubicin concentrations (1 and 0.3 µM) for 48 h and followed for next up to 6 days. Exposure of hiPCS-CMs to 1 µM doxorubicininduced suppression of both cardiac troponin T (cTnT) and cardiac troponin I (cTnI) gene expression. Conversely, lower 0.3 µM doxorubicin concentration produced no significant changes in the expression of aforementioned genes. However, the intracellular topography, arrangement, and abundance of cardiac troponin proteins markedly changed after both doxorubicin concentrations. In particular, at 48 h of treatment, both cTnT and cTnI bundles started to reorganize, with some of them forming compacted shapes extending outwards and protruding outside the cells. At later intervals (72 h and onwards), the whole troponin network collapsed and became highly disorganized following, to some degree, overall changes in the cellular shape. Moreover, membrane permeability of cardiomyocytes was increased, and intracellular mitochondrial network rearranged and hypofunctional. Together, our results demonstrate complex effects of clinically relevant doxorubicin concentrations on hiPCS-CM cells including changes in cTnT and cTnI, but also in other cellular compartments contributing to the overall cytotoxicity of this class of cytostatics.

## 1. Introduction

Chemotherapy-related cardiotoxicity continues to be one of the limiting factors in the antineoplastic treatment regimens leading to a significant damage of the heart with consequent cardiac failure in treated patients [1]. One of the cytostatic drugs that is often implicated in acute as well as chronic cardiotoxicity is an anthracycline antibiotic (ANT) doxorubicin (DOX). Traditionally, the ANT (DOX)-induced cardiotoxicity and resulting chronic heart failure have been related to iron-catalyzed oxidative damage to the heart [2]. However, recently it has been suggested that the cardiotoxicity is triggered by an interaction of ANT with topoisomerase II beta (TOP2B) in cardiomyocytes [3], thereby suggesting a more complex set of mechanisms behind ANT-related toxic effects. As well as not entirely elucidated mechanisms of ANT-dependent cardiotoxicity, scientists do not thoroughly agree upon established markers of such toxicity. 

The first studies of Herman and Adamcova [4,5] in the nineties describe a significant elevation of cardiac troponin T (cTnT) in experimental models of chronic ANT cardiotoxicity. Later, numerous experimental papers have demonstrated that ANT-induced cardiac damage is associated with a relatively prolonged release of cardiac troponins (cTns) related to the cumulative dose of ANT and that cTns are very important biochemical markers for evaluation of cardiotoxic or potentially cardioprotective effect of new agents [6,7,8,9,10,11]. 

A significant increase of cTns after ANT exposure has been also found in numerous clinical studies. When measured as an early biomarker of cancer cardiotoxicity, cTns rise occurs consistently in 21–40% of patients after anthracycline chemotherapy, irrespective of assay type. Though cTnI and cTnT levels are not directly comparable across the available assays, representative levels ranged from 11 to 120 ng/L in low-dose, and 160 to more than 1900 ng/L in high-dose anthracycline regimens [12].

Moreover, many clinical studies have also demonstrated that both cTnI and cTnT indicate cardiotoxicity before any decrease in left ventricular ejection fraction (LVEF) has occurred in patients treated with ANT therapy [13]. Recently, Cardinale et al. [14] have highlighted that the use of troponins enable i) prediction of severity of future left ventricular (LV) dysfunction, because troponin c_max_ after chemotherapy correlated closely to reduction of LVEF; ii) cardiac risk stratification after chemotherapy, which allows for personalization of the intensity of cardiac monitoring; iii) better patients selection for cardioprotective therapy; iv) most patients exclusion from long-term cardiologic follow-up [15,16,17,18]. 

Therefore, cTns have been proposed to be a more sensitive surrogate for the detection of ANT-associated cardiotoxicity than the currently recommended method of monitoring left ventricle systolic function [12,19,20]. Despite lacking evidence of the particular mechanisms of cardiac cTns release, many researchers have adopted these measurements into ANT monitoring protocols and the European Society of Medical Oncology has published guidance for cTnI monitoring in patients exposed to ANT [18]. The ongoing UK multicenter Cardiac CARE study (ISRCTN24439460) aims to stratify ANT-treated patients according to high sensitivity cTnI measurement and the patients exhibiting high-risk will be randomized to cardioprotection therapy [20]. 

Irrespective of these guidelines and practices, several issues persist such as the question of the optimal timing of patient blood sampling. Our previous study described for the first time the diagnostic window of cTns in the development of chronic ANT cardiotoxicity, which differs significantly from acute myocardial infarction. On the well-validated model of chronic daunorubicin cardiotoxicity, the plasma levels of cTns progressively increased with the cumulative dose of chemotherapy. The significant rise occurred with a peak between 4–6 h and a declined until 24 h. Discrete cTns release continued even after cessation of the therapy [21]. 

Still, despite the extensive research on cTns release as a biomarker of cardiotoxicity, the possible mechanisms behind cTns elevation in all clinical settings (including ischemic/reperfusion injury) have not been completely understood. Moreover, the data about the expression and morphological alterations of cTns in DOX-exposed cardiomyocytes related to their possible release are missing too. Hence, the present study was designed to investigate dynamics of acute and delayed response of human cardiomyocytes derived from human induced pluripotent stem cells (hiPSCs) to DOX. The special emphasis was placed on morphological aspects of exposed cardiomyocytes together with observations into select established DOX targets such as cTns as well as mitochondria.

## 2. Results

### 2.1. iCell Cardiomyocytes Maintenance and Viability

**Figure 1:**
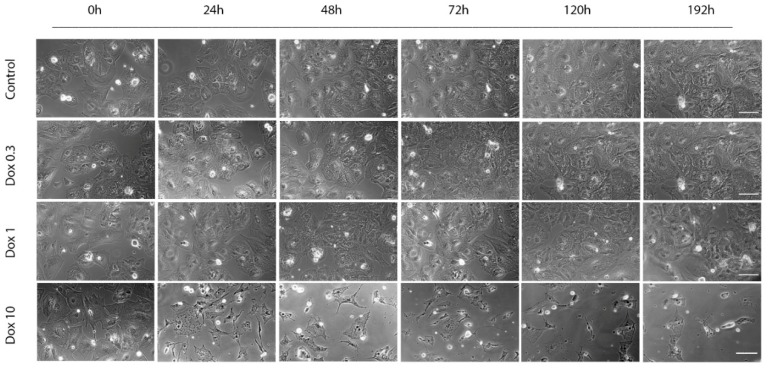
Time-lapse videomicroscopy of induced pluripotent stem cells (hiPCS-CMs) exposed to a range of doxorubicin (DOX) concentrations (10, 1 and 0.3 µM) during 192 h. Control and DOX-exposed hiPCS-CMs cultures maintained according to the manufacturer’s recommendation were continuously screened with enhanced phase contract microscopy using BioStation IM system over the mentioned time intervals. Resulting time-lapse sequences were adjusted and frame by frame analyzed using specialized software. Shown are representative morphologies of cardiomyocytes at individual time intervals. Phase contrast 400×. Bar 30 µm.

The hiPCS-CMs (Cellular Dynamics International, Madison, WI, USA) obtained from the frozen vials according to manufacturer’s instructions showed viability of minimum 88%. After their plating on fibronectin-treated surfaces of coverslips or cultivation vessels (plates) hiPCS-CMs assumed typical morphology of large adherent myocytes which gradually formed a beating monolayer with morphologically and spatially heterogeneous cells. The appearance and external behavior of these cells (i.e., beating) did not change during the entire experiment (up to 192 h of observation—Figure 1). 

**Figure 2:**
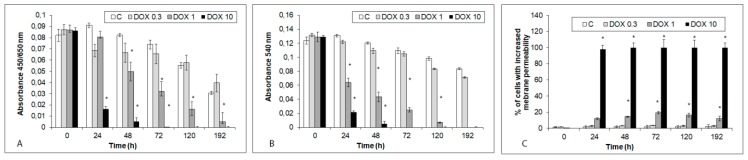
Effects of DOX (10, 1 and 0.3 µM) on viability, metabolism and membrane permeability of hiPCS-CMs during 192 h has measured by WST-1, Neutral red (NR) and ethidium bromide assays as described in Materials and methods section. (**A**) WST-1 assay (**B**) NR assay (**C**) ethidium bromide assay. Values represent means ± SD of at least three experiments * *p* < 0.05 compared to untreated control cells at the same treatment interval with one way-Anova test and Dunnett’s post test for multiple comparisons.

Viability of hiPCS-CMs in time was measured by three independent approaches; by WST-1 assay which determines the glycolytic production of NAD(P)H, by neutral red (NR) assay focused on ability of viable cells to incorporate and bind the supravital dye NR in the lysosomes and by ethidium bromide assay measuring the integrity of cellular membranes. Metabolic activity of control hiPCS-CMs decreased in a time-dependent manner, with a significant drop detected at the end of the study (192 h) (Figure 2A). Conversely, while generally decreasing, an overall ability of cells to uptake NR and retain it within lysosomes as measured by NR content in the cells was not significantly reduced throughout the study. Similar results were obtained with ethidium bromide permeability inside the cells (Figure 2B,C).

### 2.2. Acute and Delayed Doxorubicin Effects on iCell Cardiomyocytes Morphology and Viability

Exposure of hiPCS-CMs to DOX-induced concentration and time-dependent changes in their morphology, behavior, and viability. The highest DOX concentration of 10 µM had a rather rapid and overwhelming effect on the cells. Already at 24 h of exposure most cells either lost their adherence to substratum, rounded up and became disintegrated or underwent fast intracellular degeneration which left some cells over-extended and fixed. Very few cells exhibited apoptotic morphologies—i.e., shrinkage and membrane blebbing. No further changes occurred at later time intervals and individual cells gradually fragmented. Loss of hiPCS-CMs viability and increased membrane permeability as measured by the employed assays showed corresponding dynamics; i.e., from 24 h of exposure onwards cells significantly stopped their metabolic activity and their membranes became fully damaged (Figure 2A–C). 

Both 1 and 0.3 µM DOX concentrations had less marked effects on both morphology as well as functionality of exposed cardiomyocytes as evident from their appearance and performance in the mentioned tests. Still, the extent of DOX-associated damage was concentration-dependent and the altered assay parameters appeared in varying time intervals; in case of 1 µM DOX loss of viability appeared between 24 and 48 h of exposure and continued until the end of the experiment even after removal of DOX. Conversely, while continuously reducing cells metabolism and viability in time 0.3 µM DOX-dependent cytotoxicity has never become significant at any observed time interval (Figure 1 and Figure 2B,C).

### 2.3. Modes of iCell Cardiomyocytes Damage after Acute and Delayed Doxorubicin Exposure

**Figure 3:**
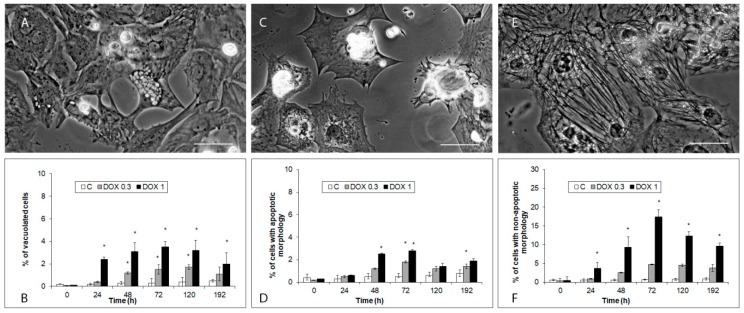
Effects of 1 and 0.3 µM DOX on appearance of select morphologies in hiPCS-CMs cardiomyocytes during 192 h. Cells were exposed to DOX and at individual time intervals, their morphology was examined and quantified by the Cell Scoring module of MetaXpress^®^ Image Acquisition and Analysis Software. (**A**,**B**) Cells with vacuoles. Phase contrast 400×. Bar 10 µm. *p* < 0.05 * Significantly higher than control at the same treatment interval (**C**,**D**) Blebbing cells. Phase contrast 400×. Bar 10 µm. *p* < 0.05 * Significantly higher than control at the same treatment interval (**E**,**F**) Extended cells. Phase contrast 400×. Bar 10 µm. *p* < 0.05 * Significantly higher than control at the same treatment interval one way-Anova test and Dunnett’s post test for multiple comparisons.

During the treatment of hiPCS-CMs with both DOX concentrations (1 and 0.3 µM), already at 24 h of exposure several types of altered cell morphologies appeared. Firstly, cells with vacuolated cytoplasm (either multiple smaller vacuoles or several larger ones—Figure 3A,B) where individual vacuoles stained positive for mitochondria (most) or autophagosomes (minority). Vacuolization developed individually in particular cells, showed varying dynamics and persisted sometimes until the end of the experiment. Alternatively, vacuolated cells at some time points shrank and underwent fragmentation. Secondly, rounded blebbing cells displaying characteristic apoptotic features peaking at 72 h of exposure but never comprising a significant proportion of the treated cell population (Figure 3C,D). Finally, and in most significant numbers, enlarged cells with reticular-like cytoplasm and very heterogeneous shapes and sizes. Their numbers reached maximum at 72 h of exposure and then they decreased (1 µM DOX) or stayed the same (0.3 µM DOX) (Figure 3E,F).

### 2.4. Effects of Acute and Delayed Doxorubicin Exposure on the Expression and Organization of Troponins in iCell Cardiomyocytes

**Figure 4:**
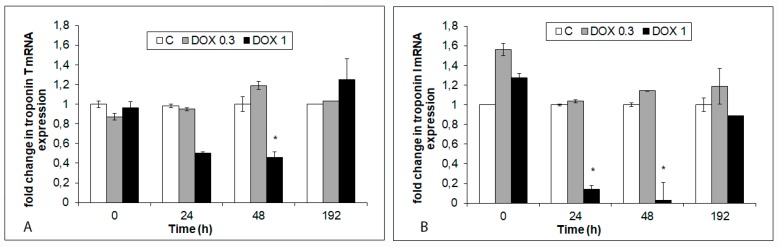
Effects of 0.3 µM and 1 µM DOX on the expression of cardiac troponin T and troponin I genes in hiPCS-CMs during 192 h. Control and DOX-exposed cardiomyocytes were harvested at individual time intervals, mRNA was isolated and its quantity determined by qPCR as described in Materials and Methods. (**A**) Expression of troponin T. *p* < 0.05 * Significantly lower than control at the same treatment interval one way-Anova test and Dunnett’s post test for multiple comparisons. (**B**) Expression of troponin I. *p* < 0.05 * Significantly lower than control at the same treatment interval one way-Anova test and Dunnett’s post test for multiple comparisons.

Exposure of hiPCS-CMs to 1 µM DOX-induced suppression of cTnT (48 h of treatment) and cTnI gene expression (24 h and 48 h of exposure). Conversely, lower 0.3 µM DOX concentration produced no significant changes in the expression of the aforementioned genes (Figure 4A,B). 

**Figure 5:**
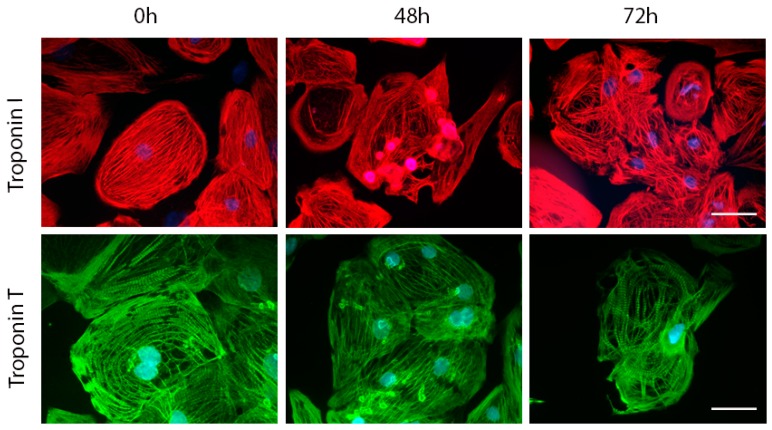
Effects of 1 µM DOX on the localization and arrangement of troponin T and troponin I in hiPCS-CMs during 72 h. Control and DOX-exposed cardiomyocytes were at individual time intervals rinsed with PBS, fixed, permeabilized and incubated with particular antibodies as specified in Materials and Methods section. Specimens were mounted and troponin T and troponin I-specific fluorescence were examined by the fluorescent microscopy and cell analysis as described in Materials and Methods. Fluorescence 400×. Bar 10 µm.

Unlike these differing effects of the employed DOX concentrations on cTnT and cTnI genes, the intracellular topography, arrangement and amount of these proteins markedly changed after both DOX concentrations. In particular, at 48 h of treatment, both cTns bundles started to reorganize, with some of them forming compacted shapes extending outwards and protruding outside the cells. At later intervals (72 h and onwards) whole troponin network collapsed and became highly disorganized following to some degree overall changes in the cellular shape (Figure 5). 

### 2.5. Effects of Acute and Delayed Doxorubicin Exposure on the Organization and Function of Mitochondria in iCell Cardiomyocytes

**Figure 6:**
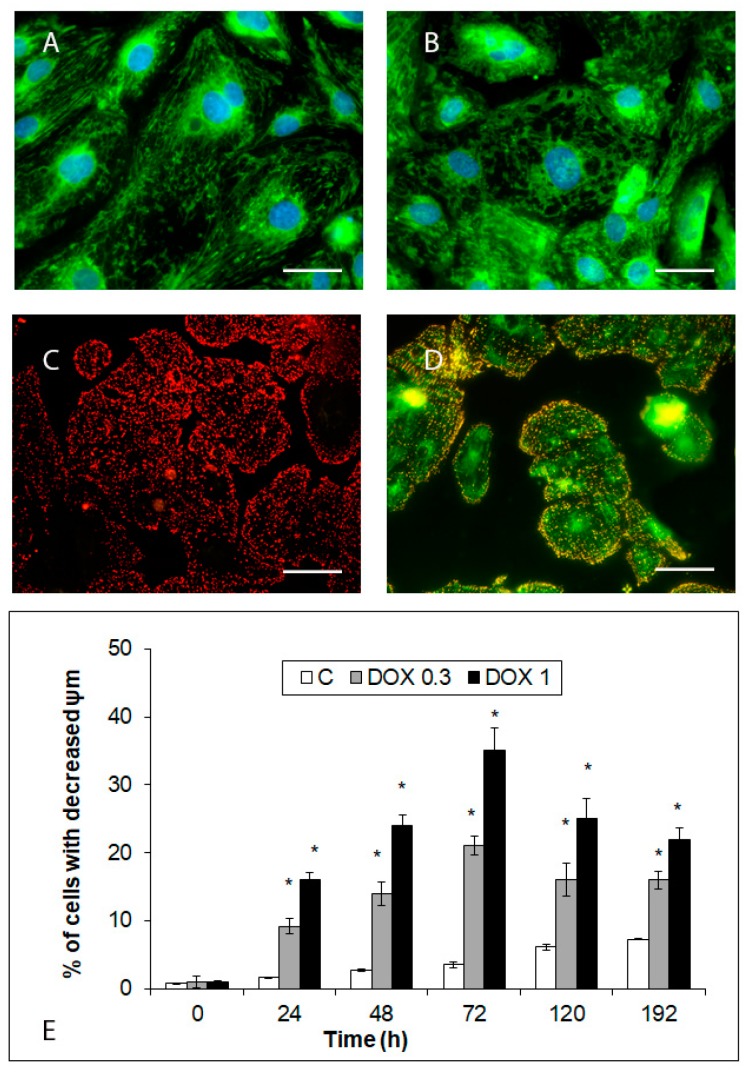
Effects of 1 and 0.3 µM DOX on mitochondrial topography and activity in hiPCS-CMs during 192 h. Mitochondrial morphology and arrangement in (**A**) control and (**B**) 1 µM DOX-treated cells (72 h) was examined by microscopic evaluation of MitoTracker Green FM fluorescence. Mitochondrial membrane potential (ψ) in (**C**) control and (**D**) 1 µM DOX-treated cells (72 h) was examined by microscopic measurement of potential-sensitive JC-1 dye. Software-aided cell quantitation of ψ in 0.3 and 1 µM DOX-treated cells during 192 h (**E**) as described in Materials and methods section. Fluorescence 400× (**A**,**B**) and 200× (**C**,**D**). Bars 10 µm (**A**,**B**), 30 µm (**C**,**D**). * *p* < 0.05 compared to untreated control cells at the same treatment interval with one way-Anova test and Dunnett’s post test for multiple comparisons.

Control hiPCS-CMs contain long, filamentous mitochondria extending throughout the entire cytoplasm where they constantly dynamically change their position and shape. Upon treatment with DOX, this dynamics temporarily increased but from 24 h of exposure lessened and eventually stopped, with individual mitochondrial clusters concentrating in the perinuclear area and in or around vacuoles. Concomitant with altered morphology, mitochondrial membrane potential in hiPCS-CMs started to drop, reaching the maximum extent at 72 h of exposure and then again increasing but remaining reduced in significant numbers of treated cells until the end of the experiment (Figure 6).

## 3. Discussion

Current studies have demonstrated that in vitro use of human embryonic stem cell (hESC)- or (hiPSC)-derived cardiomyocytes can be beneficial for preclinical safety assessment. Recent research indicates that hiPSC-cardiomyocytes are a promising tool for evaluation of both structural and electrophysiological drug-induced cardiotoxicity [22,23,24,25] as well as for searching of new biomarkers of cardiotoxicity [26,27,28,29].

In the present study, hiPCS-CMs were treated with DOX concentrations of 10, 1 and 0.3 µM for 48 h, with subsequent washout and monitoring for next up to 6 days to mimic both acute as well as delayed DOX toxicities. It is necessary to point out that we have used the doses of DOX, which are well within the clinical therapeutic range where the highest values of DOX c_max_ in plasma at the end of infusion are maximally 10 µM [30]. Also, similar concentrations are also used in reputable laboratories worldwide [11,31].

Our results show that the high DOX concentration (10 µM) induced significant changes in cardiomyocytes viability, morphology and behavior which were apparent already at 24 h of exposure and corresponded to fast necrotic death. Similar loss of viability with a concomitantly increased membrane permeability were observed in case of 1 µM DOX treatment too albeit at a different time frame and with variable final cell morpho-phenotypes. These included vacuolated cells and cells with apoptotic or non-apoptotic morphologies. On the other hand, the lowest employed DOX concentration (0.3 µM) did not significantly change previously mentioned parameters at any time interval while the same morpho-phenotypes in exposed cells were detected too. These data suggest that DOX-related cardiomyocyte damage is concentration dependent and the resulting cell phenotypes related to toxic effects of DOX are heterogeneous including their both transient and permanent appearances. 

Traditionally, ANT induced cardiotoxicity has been experimentally verified via cardiomyocyte damage-dependent loss of cTns detected in the medium as an ultimate biomarker of observed cell damage [32,33]. Once believed to originate from cardiomyocyte necrosis, it is now suggested that multiple mechanisms contribute to the cTns release including necrosis, apoptosis, normal myocyte cell turnover, release of proteolytic cTns degradation products, increased cell-membrane permeability due to integrin-mediated “stretch” mechanisms, and formation and release of membrane blebs [34]. In our previous study isolated rat neonatal ventricular cardiomyocytes NVCM were subjected to 72 h treatment with 0.1 µM, 0.3 µM, 1 µM and 3 µM of daunorubicin and culture medium was sampled at 0 h, 3 h, 6 h, 12 h, 24 h, 48 h and 72 h of daunorubicin exposure for determinations of both cTnT and cTnI. The significant increase of cTns occurred at 48 and 72 h of ANT exposure in cardiomyocytes treated with 1 µM and 3 µM of ANT only [33]. Due to the complexity of mechanisms of cTns loss and with respect to our studied heterogeneous mix of exposed cardiomyocyte phenotypes, rather than focusing on mere cTns release, this study aimed to explore changes in cTns expression, topography as well as on other relevant intracellular stress events. Firstly, 1 µM but not 0.3 µM DOX temporarily reduced the expression of cTnT and cTnI genes in exposed cardiomyocytes. This observation is in accordance with Chaudhari et al., who showed using the GO and KEGG pathway analysis that DOX exposure preferentially suppressed the expression of genes involved in cardiac contraction and pathways related to cardiomyopathies [28]. Unlike these differential gene-specific effects, however, both DOX concentrations produced significant and lasting changes in morphology and topographical organization of cTnT and cTnI proteins in exposed cardiomyocytes. The most notable ones were cTns cytoplasmic rearrangements where their regular bundling patterns were replaced with a random amassment which persisted over the entire experiment. Moreover, disturbances in the cells’ contractility and beating patterns occurred too. Similar alterations in DOX-treated human cardiomyocytes were reported before [26,35], however, without such a detailed emphasis on morphological aspects of cTns. Thus, collectively our data indicate that morphological and likely functional changes produced by DOX in human cardiomyocytes have a lasting effect and through their persistence might contribute to their delayed malfunctioning. 

It is nowadays recognized that mitochondria are among the important targets of DOX-dependent cardiomyocytes toxicity [36]. A number of studies reported acute effects of DOX on mitochondrial network structure and function associated with increased production of free oxygen and nitrogen species. In this respect, mitochondria may not only be a target of direct DOX toxicity but also contribute to the overall cell damage by inducing oxidative stress [37]. The role of mitochondrial damage in chronic (delayed) ANT-related toxicity is generally acknowledged too, with several studies reporting their employed model dependent effects of on mitochondria as well as on mitochondrial biogenesis signaling pathways [38,39,40]. Thus, is has been suggested that DOX induces fragmentation of mitochondria [41], their disorganization and functional damage [42] as well as mitochondrial enlargement and matrix disorganization [43]. The extent and permanence of such a mitochondrial damage clearly depends on the employed DOX dose too since nanomolar DOX has already been shown to cause mitochondrial disturbances [29]. Our own results confirm these observations but also show that beyond chronically disturbed mitochondrial network a significant number of followed surviving cardiomyocytes displayed reduced mitochondrial membrane potential. Although their numbers peaked at 72 h of exposure they were still markedly present by the end of the experiment (192 h) which seems to indicate that their recovery is a slow process and the possibility of permanent existence of such cells is real. 

## 4. Materials and Methods

### 4.1. Cell Cultivation, Plating, and Maintenance

Cryovials with frozen hiPCS-CMs were thawed and carefully resuspended in plating medium according to the company’s instructions. Next, viability and density of thus acquired hiPCS-CMs were determined by a trypan blue and hemocytometer (average viability rate was more than 80%). One cryovial typically yielded a 24 mL suspension of viable cardiomyocytes of an average concentration of 200,000 cells/mL. Prior (24 h before) to thawing of hiPCS-CMs, sterile coverslips (diameter 12 mm) were placed into individual wells of a series of 24-well cultivation plates. A working solution of fibronectin (1 µg/mL) was prepared from a stock solution (1 mg/mL) by addition of an adequate volume of sterile D-PBS and 600 µL of this solution was added to each well. Plates with thus treated wells were incubated at 37 °C and 5% CO_2_ for 24 h. Next day, fibronectin solution was removed from wells and immediately 600 µL of prepared hiPCS-CMs suspension was pipetted to each well (the final concentration was 120,000 cells/well). Plates with seeded hiPCS-CMs were incubated at 37 °C and 5% CO_2_ for 48 h. Next, plating medium in each well was resuspended, removed and exchanged for maintenance medium which was thereafter typically exchanged every 48 h as per recommendation of the producer until the end of the experiment and/or existence of the cultivation. 

Alternatively, viable hiPCS-CMs were plated into 96-well plates pretreated with fibronectin as explained before (the final concentration 30,000 cells/well) and their further maintenance and treatment followed the above-mentioned protocol.

### 4.2. Experimental Scheme

The hiPCS-CMs were seeded onto coverslips in 24-well cultivation plates (or 96-well plates) according to the above-mentioned procedure. Following the 48 h cultivation of cells in maintenance medium, it was exchanged with fresh DOX-free maintenance medium (controls) or maintenance medium with dissolved DOX at the tested concentrations (10, 1 and 0.3 µM). The iCell cardiomyocytes were exposed to DOX for 48 h, then DOX was removed and cells in DOX-free maintenance medium were analyzed for another 6 days using several assays. 

### 4.3. Time-Lapse Microscopy of Cellular Morphology

Morphology of control and doxorubicin exposed hiPCS-CMs was followed by a time-lapse imaging system BioStation IM (Nikon, Prague, Czech Republic) which recorded multiple cells at varying magnification and time-lapse modes (small as well as high for both global and detailed view of changes in behavior of treated cell populations). Recorded sequences were subsequently semi-automatically analyzed with the software NIS Elements AR 4.20 (Nikon, Prague, Czech Republic) and individual representative frames chosen for demonstration of detected trends.

### 4.4. Cell Viability and Proliferation

Control and DOX-treated hiPCS-CMs maintained in 96-well plates were at the particular time intervals washed with fresh maintenance medium (the old medium was removed). Next, cells were washed twice with PBS and a medium with 100 µL of WST-1 (0.3 mg/mL) was added to each well. After 2 h of incubation (37 °C, 5% CO_2_), the absorbance was recorded by a fluorimeter SPEKTRAFluor Plus, Tecan (Salzburg, Austria) at 450 nm with 650 nm of reference wavelength. In all cases, the absorbance of the tested doxorubicin in medium alone was recorded to correct for potential inference.

In a parallel assay, control and DOX-treated hiPCS-CMs maintained in 96-well plates were at the particular time intervals stained with NR (100 µL of NR at the concentration of 80 µg/mL in fresh medium) and incubation at 37 °C and 5% CO_2_ continued for 3 h. Next, the medium was removed, and cardiomyocytes were fixed in 100 µL of fixative solution (1 g/100 mL CaCl_2_ in 0.5% formaldehyde) for 15 min in room temperature. Following the fixation, cells were washed twice with PBS and lysed in 200 µL of lysis solution (1% CH_3_COOH in 50% EtOH) for 30 min. The number of cardiomyocytes released NR was measured by a fluorimeter SPEKTRAFluor Plus, Tecan (Salzburg, Austria) at 540 nm. 

### 4.5. Fluorescent Detection of Membrane Permeability

Control and DOX-exposed hiPCS-CMs were washed by maintenance medium and then stained with ethidium bromide (100 nM, 30 min, 37 °C). Next, cells were rinsed in warm maintenance medium and fluorescence resulting from ethidium bromide-positive cell nuclei was in individual cells detected by the Cell Scoring module of MetaXpress^®^ Image Acquisition and Analysis Software (Molecular Devices, LLC, Sunnyvale, CA, USA). In all experiments, on average 5000 cells (per given time interval) were included in analyses.

### 4.6. Morphological Analysis of Cell Damage

Morphologies of control and DOX-exposed hiPCS-CMs were at the specified time points analyzed the Cell Scoring module of MetaXpress^®^ Image Acquisition and Analysis Software. In all experiments, on average 5000 cells (per given time interval) were included in analyses.

### 4.7. Fluorescent Detection of Mitochondrial Membrane Potential (ψm)

At regular time intervals, coverslips with control and DOX-exposed hiPCS-CMs were rinsed in warm maintenance medium and stained with cationic JC-1 dye (10 μg/mL) for 15 min at 37 °C, 5% CO_2_. Next, medium with JC-1 was removed, coverslips were five times thoroughly but gently washed with warm medium and changes in mitochondrial membrane potential in individual cells were assessed by a fluorescence microscope Nikon Eclipse E 400 (Nikon, Prague, Czech Republic) using the software NIS Elements AR 3.20 for semi-quantitative image analysis. 

### 4.8. Fluorescent Detection of Mitochondrial Network

At regular time intervals, coverslips with control and DOX-exposed hiPCS-CMs were rinsed in warm maintenance medium and stained with MitoTracker Green FM (100 nM, 30 min, 37 °C) and Hoechst 33,528 (10 nM, 30 min, 37 °C). Thereafter, medium with flurescence dyes was aspirated, coverslips were five times thoroughly but gently washed with warm medium and mounted. Mitochondrial topography in individual cells was visualized by a fluorescence microscope Nikon Eclipse E 400 (Nikon, Prague, Czech Republic) using the software NIS Elements AR 3.20 for semi-quantitative image analysis. 

### 4.9. Immunofluorescent Detection of Troponins

Control and DOX-exposed hiPCS-CMs on coverslips were washed firstly by maintenance medium followed by pre-warm PBS and fixed with 4% paraformaldehyde (10 min, 25 °C). Next, there were rinsed with phosphate saline buffer with 1% Triton X (PBS-T), blocked in 5% BSA for 1 h at 25 °C and incubated with anti-cTnT (Abcam, 1:200) and anti-cTnI (Abcam, 1:200) at 4 °C for one hour. The cells were washed 3 times with cold PBS (each 5 min, 25 °C) and secondary antibodies (Alexa Fluor-488/568-conjugated - Invitrogen-Molecular Probes, Inc., Carlsbad, CA, USA) were added for another 1 h incubation at 4 °C. The cells were again washed 3 times in PBS (each 5 min, 25 °C) and then mounted into Prolong^®^ Gold anti-fade mount with DAPI (Invitrogen-Molecular Probes, Inc., Carlsbad, CA, USA). Images were taken with a fluorescence microscope Nikon Eclipse E 400 (Nikon, Prague, Czech Republic) and NIS Elements AR software (Nikon, Prague, Czech Republic).

### 4.10. Quantitative Real-Time RT-PCR Detection of Troponins Expression

Control and DOX-exposed hiPCS-CMs from 12-well plates (150,000 cells/well/mL) were collected using TriReagent (Sigma Aldrich, Prague, Czech Republic) and total RNA was isolated using Direct-zol RNA MiniPrep kit according to manufacturer’s instructions (ZymoResearch, Irvine, CA, USA). RNA yields and purity were determined measuring the absorbance at 260 and 280 nm using NanoDrop ND-2000 UV-Vis Spectrophotometer (Thermo Fisher Scientific, Waltham, MA, USA). Absorption ratio A260/A280 of all tested samples was greater than 1.8. The quality of RNA was checked by Agilent 2100 Bioanalyzer and the RNA integrity number (RIN) was greater than 9.0. First strand cDNA was synthesized from 1 µg total RNA using RevertAid Reverse Transcriptase according to the manufacturer’s instruction (Thermo Fisher Scientific, Waltham, MA, USA). After initial heat denaturation of total RNA (65 °C for 5 min), the reactions (20 µL) were incubated for 10 min at 25 °C, for 50 min at 42 °C and for 5 min at 80 °C. Obtained cDNA was diluted (5x) prior to qPCR. All cDNAs were stored at −20 °C until qPCR assay.

The primers for mRNA quantification were designed using Primer3. All primers were synthesized by GENERI BIOTECH (Hradec Kralove, Czech Republic). The specificity of the primers was checked by NCBI Blast tool and the reaction conditions were optimized by determining the primer concentrations. The sequences of primers were as follows: h_cTnI-F (CGTGTGGACAAGGTGGATGA), h_cTnI-R (GCCGCTTAAACTTGCCTCG), h_cTnT-F (GGAGGAGAACAGGAGGAAGG) and h_cTnT-R (CTGGATGTAACCCCCAAAATGC). The qPCR analyses were performed in Corbett RotorGene PCR Detection System using SYBR Green I detection in a final volume of 20 µL. The reaction mixture consisted of components from Fast Start Universal SYBR Green Master (Rox) (Roche Life Science, Mannheim, Germany) as specified by manufacturer, both forward and reverse primers (final concentrations 100 nM), and 10 µL of diluted cDNA. The PCR reactions were initiated by the denaturation step of 10 min at 95 °C, followed by 40 cycles of amplification, which were performed according to the following thermo cycling profiles: denaturation for 10 s at 95 °C and annealing and extension for 40 s at 60 °C. Fluorescence data were acquired during last step. Dissociation protocol with a gradient (0.5 °C every 2 s) from 65 °C to 95 °C was used to investigate the specificity of the qPCR reaction and presence of primer dimers. Gene-specific amplification was confirmed by a single peak in the melting curve analysis. The size of all amplicons was confirmed by 2% agarose gel electrophoresis stained with SYBR Safe DNA gel stain (Invitrogen, Waltham, MA, USA). All qPCRs were run in duplicates. Calculations were based on the “Delta–Delta Ct method”. The data were expressed as fold change of the cell cultures relative to the control. Glyceraldehyde 3-phosphate dehydrogenase (GAPDH) was used as reference gene for mRNA analysis.

### 4.11. Statistical Analysis

All experiments were repeated at least three times. Data analysis was performed by GraphPad Prism (GraphPad Software version 6.0, Inc. San Diego, CA, USA). Statistical analysis was carried out using one-way analysis of variance (ANOVA) followed by Dunnett’s multiple comparisons test significant at a level of *p* < 0.05. In case of troponin I and T gene expression analyses two-way ANOVA with Tukey’s multiple comparisons test was employed. 

## 5. Conclusions

In conclusion, our results bring evidence of dose-dependent changes in the metabolism, cell membrane permeability, and cell death in human cardiomyocytes exposed acutely and chronically to DOX. DOX at clinically relevant concentrations temporarily downregulated the expression of cTnT and cTnI genes and induced structural and functional changes in both cTns. In addition, in thus exposed cells, chronic changes in the topography of mitochondrial network, as well as the reduction of mitochondrial membrane potential, took place, which could be responsible ultimately for the cardiomyocyte malfunction and failure.
